# Online food allergy and anaphylaxis education for school personnel is effective and scalable: experience with the allergyaware e-learning portal from 2015 to 2022

**DOI:** 10.1186/s13223-025-00977-0

**Published:** 2025-07-06

**Authors:** Bhanu Sharma, Stephanie Ayers, Joni Huang, Jennifer Gerdts, Susan Waserman, Anthony J. Levinson

**Affiliations:** 1 McMaster University, 1280 Main Street West, Hamilton, ON L8S4L8 Canada; 2 Food Allergy Canada, 507-505 Consumers Rd North York, Toronto, ON M2J 4A2 Canada

**Keywords:** Food allergy, Anaphylaxis, Anaphylaxis awareness, Anaphylaxis prevention, E-learning, Web-based education, Online training, Digital health, School health, School personnel

## Abstract

**Background:**

Providing training to school personnel on how to prevent, recognize, and manage anaphylaxis is critical. Asynchronous e-learning (where instructional material is available on-demand) may be well-suited to help with the implementation, scale, and dissemination of this training. In this paper, we report on the design, pilot testing, widespread implementation, and evaluation of an online course for school personnel.

**Methods:**

Best practices in evidence-based instructional design for e-learning were incorporated into the course. Content was based on consensus guidelines and reviewed by experts. Initial pilot testing demonstrated large gains in post-course knowledge and self-confidence, as well as several suggestions for improvement. A website was developed to facilitate self-enrolment and delivery of the course modules in 2015 and has since been freely available.

**Results:**

From 2015 to 2022 there have been over 170,000 course completions, with over one quarter of users completing the course more than once. Most completions occur at the start of the school year in the month of September. The median time to completion was about 25–30 min. Post-course participant self-reported confidence with epinephrine auto-injectors (EAIs) and emergency management of anaphylaxis was very high, and over 95% of participants would recommend the course to others. The average score on the 10-item post-course quiz was 9.05, with a pass rate of over 95%.

**Conclusions:**

The AllergyAware e-learning course for school personnel is highly satisfactory to users, with high post-course quiz performance and self-reported confidence in anaphylaxis management. It provides a scalable and accessible approach to training school personnel on this critical topic. Future research is needed to assess participants’ application of knowledge in real-world settings.

**Trial registry:**

The Hamilton Integrated Research Ethics Board reviewed the study protocol and granted exemption from full review per their review process, as this was considered a quality improvement initiative.

## Background

National epidemiological survey data as of 2019 suggests that 6.7% of Canadian children have either a self-reported or physician-diagnosed food allergy [1]. These estimates, however, may soon become conservative given that the prevalence of self-reported food allergy has risen in recent years [2, 3, 4]. In addition to the risk of allergic reactions and anaphylaxis associated with food allergies, children can also face concurrent psychosocial burden; childhood food allergies require daily management and are associated with reduced quality of life [5, 6, 7], food-related anxiety and stress [8, 9, 10], social stigma [11], and peer bullying [12, 13, 14].

More severe allergic reactions to food, such as anaphylaxis, are experienced by upwards of 40% of children with food allergies [3]. Anaphylaxis is a multi-system hypersensitivity reaction characterized by a rapid onset of symptoms including swelling, itching and redness of skin, hypotension, and constriction of airways that can be life threatening [15]. A large multi-center cohort study involving 131 pediatric intensive care units in North America reported that food is the most common cause of anaphylaxis in children, with peanuts and milk being the most common food-related allergens [16]. In terms of high-risk settings for anaphylaxis, a multi-center Canadian study shows that 12.8% of reactions occur at school or daycare [17]. In line with this, a recent systematic review identified that there are a disproportionate number of food-induced anaphylaxis fatalities in school settings [18].

Training school personnel in the prevention, recognition, and management of anaphylaxis is therefore critical, given that intervention with epinephrine must occur promptly following a reaction [19]. Yet, research shows that the pre-hospital use of epinephrine to treat anaphylaxis is quite low at 20.98% [20]. School-related legislation, such as Sabrina’s Law in Canada and the Food Allergy and Anaphylaxis Management Act in the United States, has been introduced to try to ensure that anaphylaxis prevention and management strategies are in place in schools. Part of these multi-faceted strategies introduced by legislation involve training school personnel on recognizing the symptoms of anaphylaxis and learning how to respond to emergency situations, which may also involve the use of stock epinephrine auto-injectors (EAIs) (devices not prescribed to anyone that can be used in an emergency) [21]. School boards with a legislated anaphylaxis prevention and management strategy have demonstrated benefits including school personnel possessing a better technique for administering epinephrine, although additional training to increase awareness of anaphylaxis procedures among school personnel and parents remains recommended for all school boards [22]. Other anaphylaxis training programs have demonstrated effectiveness in improving anaphylaxis knowledge and self-efficacy [23, 24], though the in-person training approach used limits the ability to scale the training to reach a high volume of learners.

E-learning is well-positioned to provide anaphylaxis training to school personnel given that such programs can provide access to up-to-date anaphylaxis management information, be accessed at the users’ convenience, incorporate high-quality instructional design, be consistently delivered, and be scaled and spread easily to new jurisdictions [25]. To this end, we developed AllergyAware, an online, asynchronous (self-paced and on demand) anaphylaxis training program, with a version of the course specifically developed for school personnel in both English and French.

The overall aim of this study is to describe the development, implementation, and evaluation of the AllergyAware e-learning course as a training resource for anaphylaxis education. Part 1 will report on the initial design and development of the online course, along with results from our pilot testing. Part 2 will describe course uptake, user engagement, post-course quiz performance, and self-reported confidence levels, along with survey feedback and key content and technical updates made to the platform over time.

## Methods

### Initial instructional design and development, and pilot testing

#### Initial design and development phase

*Project team.* The original AllergyAware team consisted of approximately 20 individuals, including those with multi-disciplinary expertise in anaphylaxis education and health-related e-learning and instructional design, members of the target audience (including school board members and personnel such as teachers and educational assistants), and allergy/immunology medical specialists. This latter group reviewed content regularly to ensure alignment with current evidence-based, best-practice guidelines. Team members represented faculty from McMaster University and members of the Canadian Society of Allergy and Clinical Immunology, as well as content experts and representatives from Food Allergy Canada (known at that time as Anaphylaxis Canada).

*Instructional design.* Instructional design is the design and development of learning content based on proven cognitive processes intended to maximize learners’ engagement, information retention, and knowledge transfer [26]. The development of AllergyAware followed two iterative design models, namely ADDIE (Analysis, Design, Development, Implementation, and Evaluation, a framework within the Instructional Systems Design model) and the ‘Pebble-in-the-Pond’ approach, which identified early on some of the actual content and ‘whole tasks’ to be taught [27, 28]. Design and development were also informed by best practices in multimedia learning, incorporating evidence-based principles outlined by Clark and Mayer [29, 30]. Several iterations of AllergyAware were developed and refined based on user testing and feedback from experts, until a version suitable for pilot testing was ready.

#### Content development

Content was based on the consensus best practice guidelines “Anaphylaxis in Schools and Other Settings” from the Canadian Society of Allergy and Clinical Immunology [31]. Previous educational materials were adapted for e-learning, and new scenario-based e-learning and multimedia were instructionally designed and developed using the models described above. As many schools and school boards intended to use the course as required compliance training for personnel, we were also mindful with respect to incorporating key content domains related to school policies and procedures, as well as emergency management principles and practices. The focus on compliance training also informed some of the design decisions related to duration of the course, as most schools were not devoting more than an hour to their current training practices; often using an in-person ‘lunch-and-learn’ type of approach. The initial version of the course was developed using the Articulate Presenter authoring tool with subsequent iterations using a custom-developed solution. The course consisted of three required components: the main content module, a post-module survey, and a content knowledge quiz.

#### Technical infrastructure

Initial pilot testing was hosted on the Articulate Online learning management system, with subsequent versions hosted on the AllergyAware website. The website is a heavily modified version of the WordPress web content management system, with a customized database for tracking user progress and completion of the course modules. Videos within the course are hosted on the Wistia and Vimeo video hosting platforms; quizzes and surveys integrate the third-party WordPress plug-in Gravity Forms. The French version of the course uses the WordPress Multilingual (WPML) plug-in. The site and course are hosted on the Amazon Web Services (AWS) cloud platform, with the ability to scale up to large numbers of simultaneous users as needed. Initial funding for the development and pilot testing was supported by a grant from the AllerGen NCE Inc, the Allergy, Genes and Environment Network, a national research network funded by Industry Canada through the Networks of Centres of Excellence of Canada program. Subsequent funding has been provided by charitable donations and in-kind contributions. There is no cost for users to access the course.

#### Pilot testing

After the initial design and development phase, we conducted pilot testing from November 2009 to January 2010 with educators, administrators, and other school personnel in Alberta, Canada. The testing involved a pre- and post-test design with approximately one-hour of anaphylaxis education (covering topics such as general knowledge, school-based prevention strategies, and how to use EAIs; (see Table 1) using our online course. Anaphylaxis knowledge and self-reported confidence were assessed using a series of questions (11 graded items at pre-test with 9 analogous (but not identical) graded items at post-test) developed through an expert consensus process. The primary outcomes for the pilot test were related to feasibility, user acceptance, and detailed feedback/suggestions in order to make improvements to the course before broader launch and dissemination.

**Table 1:** List of content included in our piloted educational program

Program Section	Sub-Topics
Policy and legal issues	• Anaphylaxis policies • School plans and individual emergency plans
Understanding anaphylaxis	• Identify signs and symptoms • Causes • Recognize an anaphylactic emergency
How to respond to an anaphylactic emergency	• Emergency protocol • How to use an auto-injector
Reducing risk: Your allergy-safe school	• Understand “allergy safe” • Identify risk situations
Resources	• Additional links and documents for further information

### Part 2: Implementation, dissemination, and uptake

Following the design, development, and pilot testing phase, AllergyAware was prepared to be released more broadly to school personnel. Deployment involved leveraging our existing network, which included ministries of education, school boards, schools, educational associations (such as the Canadian School Boards Association), as well as Food Allergy Canada’s schools distribution lists. Further, we made a concerted effort to promote AllergyAware outside of our existing network by means of email communications, digital advertising, educational events, direct mail, and social media. This led to the uptake by additional school boards and schools across Canada, with certain school boards requiring regular (typically annual) course completion by their personnel. Seven provincial/territorial governments supported the initiative through either their Ministries of Education and/or Health, and some referenced AllergyAware as a resource on their websites.

Upon registration, user data is collected, including: first name, last name, email, and optionally, province of employment, job title, organization name, and phone number. Data were collected from July 1 to June 30 (the “academic year”) of each year from 2015 to 2022, with the exception of the 2021 cohort (where data were collected from August 1, 2021 to January 31, 2022) and the 2015 cohort (where data collection began on November 4, 2015). The pre-test component was removed during this widespread dissemination phase for practical reasons, to reduce the time commitment needed to complete the course. As part of the course completion requirements on AllergyAware, a feedback survey and post-course quiz were administered after finishing the main e-learning module; a passing score of at least 70% was required to pass the quiz and ‘unlock’ the certificate of completion for downloading. The ten questions that comprised this quiz were mapped to the course objectives and focused on understanding participant knowledge of anaphylaxis prevention and management, ability to apply that knowledge in scenario-based items, as well as awareness and use of different EAIs. As part of the feedback survey, participants were asked to complete a 5-item questionnaire evaluating their self-confidence rating with respect to EAIs and management of an anaphylactic emergency, as well as impressions of the course impact. Users also had the option to leave open-text feedback related to their experience with AllergyAware.

### Part 3: Ongoing development and annual review

The initial developmental phase was iteratively intensive, where multiple versions of AllergyAware were developed prior to finalizing the released version. However, ongoing development and an annual review of AllergyAware continued to occur to ensure that content continues to meet current evidence-informed guidelines. These annual reviews were aimed at: (1) ensuring alignment of course material with evidence-based guidelines, best practices, expert clinical consensus, and wording from Health Canada; (2) updating the course based on user feedback and changing EAI availability; and (3) integrating any technical infrastructure upgrades. For example, in adherence with the Accessibility for Ontarians with Disabilities Act (AODA), we conducted a comprehensive review of the course, website, and associated resources using a reputable accessibility checklist software to ensure conformance with Web Content Accessibility Guidelines (WCAG 2 Level AA).

## Results

### Part 1: The allergyAware course and website

**Fig. 1:**
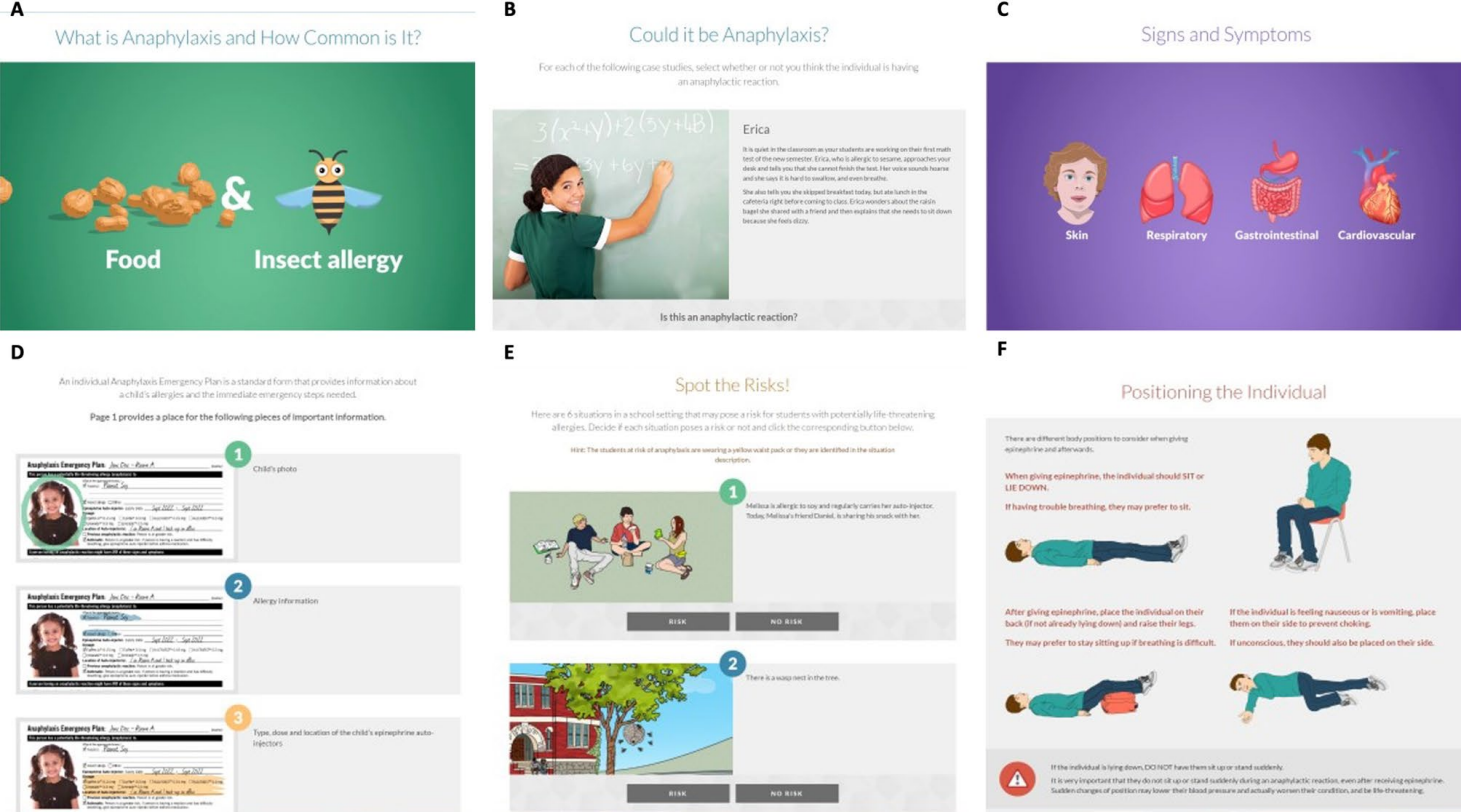
A selection of screenshots showing multimedia educational content in the allergyaware anaphylaxis in schools course. **(A)** A screenshot from the first video entitled *What is Anaphylaxis and How Common is It?* depicting two leading causes of anaphylaxis. **(B)** Use of a vignette to assess whether the scenario presented is an anaphylactic reaction. **(C)** A screenshot from the second video entitled *Signs and Symptoms* showing the body systems that can be impacted by anaphylaxis. **(D)** Step-by-step information on the components of an individual anaphylaxis emergency plan. **(E)** Scenario-based interaction wherein learners are asked to identify risk factors for an anaphylactic reaction. **(F)** Instructional graphics on how to position an individual when giving epinephrine to treat anaphylaxis (and how to keep them positioned afterwards)

The AllergyAware course (accessible via allergyaware.ca) incorporated best practices in multimedia learning using a combination of text, images, and videos to deliver educational content, as demonstrated by the screenshots in Fig. 1. Real-world scenarios and authentic case studies and resources were also used as worked examples and formative practice exercises. These images provide a representative overview of the types of multimedia presented in the main content module of the course, titled Anaphylaxis in Schools.

### Part 2: Results from pilot testing (November 2009 – January 2010)

During pilot testing, we enrolled 105 volunteers, with 74 individuals (representing elementary and secondary school teachers and administrators, educational assistants, librarians, and custodial staff) completing both assessments. Significant improvements were observed with respect to anaphylaxis knowledge (means = 51.1%, SD = 15.8 for pre-test vs. 78.2%, SD = 16.2 for post-test; F_(1,66)_ = 27.7, p < 0.001; Cohen’s *d effect size =* 1.7). With respect to specific items on the assessments that users had challenges with, the items that involved a drag-and-drop sequencing format related to the multiple sequence of steps for using the auto-injectors caused many users technical challenges with respect to having to scroll. Performance was very poor on these 3 items on the pretest with only 3%, 15%, and 18% correct responses. Although performance did improve substantially on for those items on the post-test (49%, 70%, and 80%), they continued to give many users technical issues that likely interfered with their reliability as assessment items. Two other items demonstrated poor performance on the pretest, with analogous items improving on the post-test. One was about anaphylaxis causes and the other related to the different ‘plans’– student anaphylaxis emergency plan and school plan. Most items were not directly duplicated between pretest and post-test, to avoid too much of a ‘priming’ effect on the user. The quizzes were also designed to be more ‘formative’, with feedback provided to the user to support their learning experience.

There were also improvements with respect to participant confidence in using an EAI using a 3-point Likert scale (“Not at all confident”– ”Somewhat confident”– “Very confident”), including both the EpiPen^®^ (χ^2^ = 20.2, *p* < 0.0001) and Twinject^®^ (χ^2^ = 24.0, *p* < 0.0001) EAIs, which were both represented in the initial version of the course. (Note: the Twinject device was later removed from the course as it was discontinued in the Canadian market.) In line with this, the number of participants who reported very high confidence in using the EpiPen^®^ and Twinject doubled and tripled, respectively, from pre-test to post-test (see Table 2).

**Table 2:** Program starts, completions, and percent completions, by year for the allergyaware anaphylaxis in schools course.^1^

	Starts	Completions	Percent complete
2015	18,385	17,038	92.7
2016	13,095	12,176	93.0
2017	9475^2^	8730	92.1
2018	14,605	13,587	93.0
2019	24,055	22,873	95.1
2020	47,134	44,902	95.3
2021	39,304	37,159	94.5

^1^For simplicity of reporting and data analysis, and due to more widespread adoption of the English version, only data related to the English version of the course for schools has been reported

^2^A large school board within the province of Ontario migrated to an internal anaphylaxis education program this year, thereby accounting for the decrease in program starts and completions

Results of the extensive post-course feedback questionnaire revealed general agreement and positive feedback related to usability, design, content, and various specific sections of the course. However, there were several key suggestions for improvement. First, 55% of participants disagreed with the statement that they were able to complete the program in a reasonable period of time, with several respondents noting in open text feedback that the course took too long, and the narration was too slow. Second, many participants noted frustrations with the technical aspects of the ‘drag-and-drop’ format of those assessment items related to sequencing the steps for the auto-injectors. Finally, stakeholders such as school board decision makers noted that most compliance training courses did not include pretests, in order to reduce overall duration.

The results of the pilot testing led to changes that were implemented to reduce the duration of the course and improve usability prior to widespread implementation and dissemination, including reduced audio narration, elimination of the pretest, and changing the format of the problematic assessment items.

### Part 3: Uptake and impact of allergyAware (2015–2022)

#### Course uptake

The number of times per year the course was completed after the initial pilot testing is reported in Table 3.

**Table 3:** EAI confidence levels pre- vs. post-course

Device	Confidence Level	Pretest	Post-Test	Change
EpiPen^®^	Not at all	13%	1%	-12%
	Somewhat	54%	36%	-18%
	Very	33%	63%	+ 30%
Twinject^®^	Not at all	35%	7%	-28%
	Somewhat	50%	49%	-1%
	Very	15%	44%	+ 29%

Over the 7 years the course has been offered, more than one quarter of users completed the course more than once (i.e., in different years), while 72.3% of users completed the course a single time. See Table 4 for a distribution of the number of completions of the AllergyAware Anaphylaxis in Schools course since inception.

**Table 4:** Distribution of the number of times users have completed the course from 2015–2022.^1^

Number of Times Course was Completed	Number of Users
1	88,900
2	21,200
3	4,400
4	1,400
5	4,000
6	2,700
7	300

^1^English version only

Most participants who completed the English course were from the province of Ontario (65.3%), followed by Alberta (16.7%) and British Columbia (12.5%), with lower participation (< 2.5%) from the remaining provinces and territories. (These three provinces also had the greatest percentage of repeat starts when considering only provinces and territories in which there were > 1500 repeat starts.) For the French course, 41% were from New Brunswick, 38% from Québec, and 15% from Ontario, with much smaller numbers from the other provinces and territories.

#### Time to completion

Estimates of the time to complete the course were taken from both server logs and website analytics. Average session duration for non-bounce visits (i.e., those who remain on the site after visiting the home page) was 23 min and 38 s, consistent with server-based estimates.

#### Post-module survey

Upon completing the AllergyAware Anaphylaxis in Schools course, participants were asked to complete a brief survey. This 5- or 6-question survey (depending on the year it was administered) is focused on participant self-reported confidence with EAIs/emergency management of anaphylaxis, their thoughts on the applicability and usefulness of the course, and whether they would recommend it to others. The structure of this survey remained consistent over time, though the individual items changed to reflect the available EAIs discussed in the course each year. Users are required to complete the survey in order to complete the course.

**Fig. 2:**
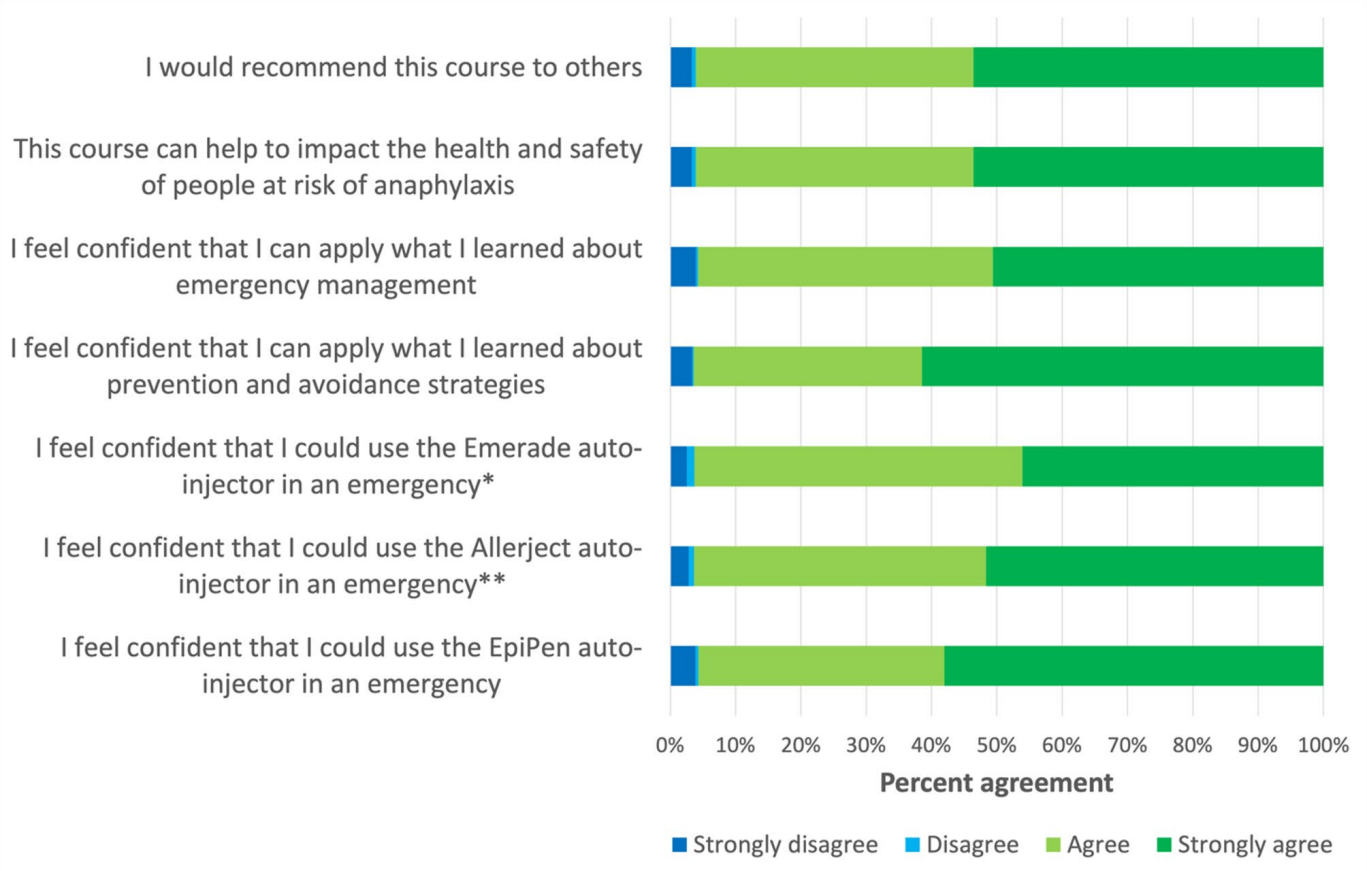
Post-course survey responses for the allergyaware anaphylaxis in schools course, with data aggregated across all years studied (2015–2022). ^*^Question was only administered in the 2021 survey. ^**^Question was only administered in the 2015, 2020, and 2021 surveys

Overall, > 95% of 170,954 participants responded with “Agree” or “Strongly agree” to each of the self-confidence items (see Fig. 2).

Open text survey comments were overwhelmingly positive, however a small number of respondents felt that the course content would be better delivered through in-person training rather than online. Several commented that they did not have access to a trainer device to practice with.

#### Content knowledge quiz

In addition to the post-module survey, participants were asked to complete a 10-item content knowledge quiz. Like the survey, the overall structure of this quiz remained consistent year-over-year, although select questions changed to reflect relevant EAIs. The passing score was set at 70%. Across all years, the average score on the 10-item quiz was 9.05 (SD = 0.18), with a modal score of 10; the pass rate per quiz attempt was 95.73% ± 1.16. The items with the poorest performance were related to ordering the steps of the EAIs. There was also an item where the user had to distinguish between a reaction being to a particular *protein*, rather than a *food*, which users had some difficulty with. Scenario-based items related to recognition of anaphylaxis or whether they would administer epinephrine were consistently correct.

## Discussion

This report detailed our experience in designing, developing, testing, implementing, and evaluating the impact of the AllergyAware online anaphylaxis course for school personnel. The first part of our report focused on methodological considerations related to developing AllergyAware, including content generation and instructional design for asynchronous e-learning. The second part centered on understanding the uptake and impact of this online anaphylaxis education program on participant self-confidence and content knowledge in the 7 years from 2015 to 2022.

Overall, we found that AllergyAware is highly satisfactory to users, who demonstrated high levels of confidence and post-test performance upon course completion. There has been steady growth in usership each year since 2015 (except for 2017, when a large school board in Ontario opted for an in-house anaphylaxis education program). This, in addition to repeat completions and survey feedback, suggests that the individual user and school board/organization experience has been positive, likely due to being conducive with their goals of ‘compliance training’– an aspect of the AllergyAware program that some other anaphylaxis educational programs lack. We also observed the highest number of completions in 2020, demonstrating the effectiveness of our program in delivering online education during the Covid-19 pandemic and remote work contexts. Accordingly, more than 95% of users responded with “Agree” or “Strongly agree” to the statement “I would recommend this course to others” on the post-module survey. The other items on this survey were similarly well-endorsed (see Fig. 2, with > 95% of participants agreeing or strongly agreeing that they were confident in identifying and managing anaphylactic events). In comparison, the study by Poza-Guedes & González-Pérez (2021) with over 1700 participants reported that following use of their e-learning module, just over 70% of participants felt “confident in facing an unexpected food allergy situation within an educational facility” [32]. Differences in post-training self-confidence between this other e-learning module and AllergyAware may be attributable to the design and content of the intervention, as well as potential differences in participant demographics, but suggest that AllergyAware may impact participant self-confidence following approximately 30-minutes of self-directed, asynchronous training. Providing such training at scale remains a priority in the field, given that a systematic review of the literature identified gaps in anaphylaxis management knowledge among physicians, patients, and the community [33].

With respect to content knowledge, the pass rate of our content quiz exceeded 95% with an average score of just over 90%, indicating strong immediate post-course performance in anaphylaxis identification, prevention, and management in school settings. This is consistent with our initial pilot testing, where we observed significant improvements between pre- and post-course quiz results with a very large effect size of 1.7. This large effect size of e-learning for knowledge outcomes is consistent with our findings from a meta-analysis of internet-based learning in the health professions [34]; it is also consistent with previous research involving online allergy education courses administered to school personnel, which demonstrated improved awareness and recognition of anaphylaxis [25]. While the content knowledge outcome measures used across various other studies differed, all programs reported post-training improvements [23, 25]. The use of evidence-based principles of instructional design for multimedia learning has been shown to improve learning outcomes [28, 29], which may account for our findings. In line with this, the design, content, and impact of the course was recognized by two awards from The Institute for Performance and Learning (formerly the Canadian Society for Training and Development).

A limitation of online education, identified by these studies amongst others [22, 35], is that they are not able to train or assess EAI administration proficiency in person. Per our experience, most users do not have EAI training devices available when completing the course, though their use is recommended. Previous studies have shown that training involving EAI devices was associated with better performance [36]. Complementing online education with in-person training may allow for a hybrid of large-scale education with individual in-setting training [37]; currently, clinical working groups suggest that e-learning should be used in cases where in-person training is not feasible [38]. Another limitation introduced in many studies assessing the effectiveness of online learning, including the current study, is the use of only an immediate post-course quiz to assess knowledge rather than employing a pretest, immediate post-test, and a delayed post-test that might better assess longer-term transfer of knowledge. Previous work has shown that initial improvements in preschool teachers’ knowledge and EAI performance after a single education session eroded significantly 4–12 weeks after the session [39].

Another key limitation is the lack of direct assessment of anaphylaxis management in real-world settings, as outcomes rely on self-reported proficiency rather than observed practice. While our findings indicate that participants report high confidence levels in anaphylaxis management following the AllergyAware course, prior research suggests that self-reported confidence does not always correlate with actual competence in emergency situations. Studies have demonstrated that school personnel may express confidence in their ability to recognize and respond to anaphylaxis but still struggle with key aspects of EAI administration when assessed through direct observation [40]. Similarly, research has identified discrepancies between self-perceived knowledge and objective performance in anaphylaxis preparedness among educators and school nurses [41, 42]. However, confidence may play a critical role in reducing response hesitancy, as lack of confidence has been associated with delays in EAI administration, a known risk factor for severe outcomes in anaphylaxis [43]. While our study did not measure real-world application of skills, future research should explore whether increased confidence translates into timely and effective intervention during anaphylactic emergencies.

Future studies are planned and will look at possible knowledge retention and attrition, as well as assessment of emergency management of anaphylaxis and EAI use through online and in-person simulation scenarios following the online course. Given the feedback from school boards and individuals about the importance of keeping the course duration as short as possible, any future work looking at pretest vs. post-test vs. delayed post-test and assessment of EAI skills will need to be done as part of a separate research protocol, instead of adding additional requirements and time to the version of the course that has been adopted by schools for their compliance training.

While we were able to make the AllergyAware course broadly available, we experienced challenges in achieving nationwide buy-in across all jurisdictions. For example, as indicated in Table 2, there was a decrease in participation in 2017, owing to the largest school board within the largest province in Canada (Ontario) opting to use their own educational module and maintain their data in-house. A Canadian study identified that in legislated jurisdictions there was greater integration of anaphylaxis guidelines into schoolboard policies [22]. Further, past research has identified both professional and institutional ‘silos’ as barriers to e-learning that preclude adoption of an e-learning program universally [44, 45]; this aligns with our experience that some provinces or larger school boards may have strong preferences to maintain control over the delivery of compliance training or employee course completion data. Research from the United States and Australia also demonstrates that in the school setting, anaphylaxis policies can differ from institution-to-institution [46, 47, 48], with some schools having no relevant policy or training requirements. If these findings generalize to the Canadian setting, this indicates that a lack of consistent policy that encourages or mandates the annual use of a program such as AllergyAware would make achieving universal buy-in difficult. Provincial legislation and policies can impact uptake considerably, where dedicated provincial or national support might help to scale and spread the course more effectively.

In addition, a recent systematic review of enablers and barriers to e-learning in health sciences education identified that a module that is (or perceived to be) too resource intensive is a barrier to effective e-learning [49]. While the review was performed in the context of health sciences e-learning for predominantly undergrad and post-graduate health profession trainees, it nonetheless suggests that it is important for e-learning modules not to be overly burdensome. This is particularly critical in the school setting, where there are other compliance training requirements for school employees and other personnel. Our data show that year to year, AllergyAware was completed on average in less than 30 min, and the time to completion remained consistent despite changes and/or updates to the learning module. We consider this a feasible time commitment, particularly given the post-course quiz and survey results, and adoption by many school boards. Our initial pilot version of the course was closer to 55 min in duration, which participants and school administrators identified as too long.

Overall, this study showed that the AllergyAware course is a feasible, user-friendly, and scalable solution that can be easily implemented in school settings with high post-course quiz performance and self-reported confidence in anaphylaxis management. Future research is needed to assess participants’ application of knowledge in real-world settings, including epinephrine auto-injector administration technique, long-term retention of key concepts, and impact on schools’ management of anaphylaxis.

## Data Availability

The data from the current study are available from the corresponding author on reasonable request.
